# Mucosal-Pull Induction of Lung-Resident Memory CD8 T Cells in Parenteral TB Vaccine-Primed Hosts Requires Cognate Antigens and CD4 T Cells

**DOI:** 10.3389/fimmu.2019.02075

**Published:** 2019-09-06

**Authors:** Siamak Haddadi, Maryam Vaseghi-Shanjani, Yushi Yao, Sam Afkhami, Michael R. D'Agostino, Anna Zganiacz, Mangalakumari Jeyanathan, Zhou Xing

**Affiliations:** Department of Pathology and Molecular Medicine, McMaster Immunology Research Centre, Michael G. DeGroote Institute for Infectious Disease Research, McMaster University, Hamilton, ON, Canada

**Keywords:** tuberculosis, vaccine, parenteral immunization, viral vector, prime-pull, lung, T cells, tissue-resident T cells

## Abstract

Tissue-resident memory T cells (T_RM_) are critical to host defense at mucosal tissue sites. However, the parenteral route of immunization as the most commonly used immunization route in practice is not effective in inducing mucosal T_RM_ cells particularly in the lung. While various respiratory mucosal (RM)-pull strategies are exploited to mobilize parenteral vaccine-primed T cells into the lung, whether such RM-pull strategies can all or differentially induce Ag-specific T_RM_ cells in the lung remains unclear. Here, we have addressed this issue by using a parenteral TB vaccine-primed and RM-pull model. We show that both Ag-independent and Ag-dependent RM-pull strategies are able to mobilize Ag-specific CD8 T cells into the lung. However, only the RM-pull strategy with cognate antigens can induce robust Ag-specific CD8 T_RM_ cells in the lung. We also show that the cognate Ag-based RM-pull strategy is the most effective in inducing T_RM_ cells when carried out during the memory phase, as opposed to the effector phase, of T cell responses to parenteral prime vaccination. We further find that cognate Ag-induced CD4 T cells play an important role in the development of CD8 T_RM_ cells in the lung. Our study holds implications in developing effective vaccine strategies against respiratory pathogens.

## Introduction

Pulmonary tuberculosis (TB) remains to be a leading single infectious threat to the global health (1). The only licensed TB vaccine Bacille Calmette-Guérin (BCG) has been administered via the skin to humans for more than six decades but it has failed to effectively control global TB epidemics. The past decade has seen more than a dozen new vaccine candidates entering various phases of clinical trials, which were developed to boost or replace BCG (2, 3). Of note, most of these vaccine candidates were designed for the parenteral route of application based on its safety, convenience, low cost, and ease of integrating into the current human immunization program. Indeed, the parenteral route is most widely and practically used in successful human immunization program. However, the parenteral route of TB vaccination is much less effective when compared to the respiratory route in a wide range of pre-clinical studies (4–8). These pre-clinical findings are supported further by recent clinical studies where unsatisfactory protection against TB was observed in parenterally vaccinated humans with either a virus- or subunit-based vaccine (9, 10). Thus, as our effort in developing new parenteral TB vaccine strategies continues, there is a pressing need to understand the immune mechanisms underlying the gaps in protective immunity by parenteral TB vaccination and develop immune-modulating strategies capable of filling such gaps.

Studies from us and others have identified that *Mycobacterium tuberculosis* (*M.tb*) infection-imposed innate immune suppression in the lung impedes timely recruitment of circulating T cells and generation of tissue-resident memory T cells in the lung of naïve or even parenteral-primed (vaccinated) hosts (11–18). In recent years, a number of respiratory mucosal (RM)-pull strategies including application of Ag-independent and Ag-dependent inflammatory/infectious agents have been explored in an attempt to correct such deficits in parenteral-primed hosts by rendering innate immune activation and/or facilitating the recruitment of parenteral-primed memory T cells to the respiratory mucosa (14, 19–23). In this regard, the property of RM-pull strategies has been found critical to the immunologic outcome in the lung. RM-pull strategies may lead to either trained innate immunity or innate immune tolerance in the lung (22, 24). On the other hand, while a number of RM-pull strategies are capable of mobilizing parenteral TB vaccine-primed T cells into the lung, they differ greatly in the longevity of recruited T cells and/or associated protective immunity (19, 22, 25). The tissue-resident memory T cells (T_RM_) have recently been recognized for their important role in anti-microbial host defense at mucosal sites (26–28). However, it remains poorly understood whether the T cells recruited into the lung of parenteral vaccine-primed hosts by using various RM-pull strategies will all be destined to be T_RM_ cells and, if not, what are the underpinning mechanisms. New knowledge in this regard will be critical to developing the effective strategies to enhance protective efficacy of parenteral vaccine strategies.

We have now addressed this issue by using a parenteral adenoviral vaccine-primed and RM-pull model. Two different types of RM-pull strategies involving the use of a cognate antigen preparation and a TLR ligand CpG were investigated. We find that the magnitude of lung CD8 T_RM_ responses is determined not only by the nature of RM-pull strategy but also by the timing of RM-pull after parenteral prime vaccination. Specifically, while both cognate antigens and CpG can effectively recruit T cells into the lung, only the cognate antigen is capable of robust CD8 T_RM_ cell induction. It is the most effective when RM-pull with cognate antigens was carried out during the memory phase, not the effector phase, of T cell responses to parenteral prime vaccination. We further find that CD4 T cells elicited by cognate antigens play an important role in induction of CD8 T_RM_ cells in the lung via conditioning the lung microenvironment. Our study thus provides new insights into the mechanisms of T_RM_ cell development in the setting of parenteral-prime and RM-pull vaccine strategies and has implications in developing effective vaccine strategies against TB and other respiratory pathogens.

## Materials and Methods

### Animals

Female BALB/c mice (6–8 weeks old) and CBy.PL(B6)-Thy1a/ScrJ BALB/c congenic strain carrying T lymphocyte–specific Thy1^a^ (Thy1.1) were purchased from The Jackson Laboratory (Bar Harbor, ME, USA). Mice were housed in a specific pathogen-free Level B facility at McMaster University. All animal experiments in this study were approved by the Animal Research Ethics Board of McMaster University and were performed in accordance with the approved guidelines for animal experimentation at McMaster University.

### Parenteral-Prime Immunization and RM-Pull Strategies

A recombinant replication-deficient chimpanzee adenovirus (Ad)-based TB vaccine expressing immunodominant *M. tuberculosis* antigen Ag85A (AdCh68Ag85A) was previously constructed in our lab and used to parenterally immunize animals (29). Vaccine was prepared at 1 × 10^7^ plaque-forming units (pfu) in 100 μl of PBS and injected at quadricep muscles of both legs as described previously (19, 29). For RM-pull strategy in parenteral vaccine-primed mice, 20 μg of unmethylated CpG oligodeoxynucleotides (GGGGGACGATCGTCG TCGGGGGG) or 2.5 μg of soluble Ag85 complex proteins (containing Ag85A/B/C purified from *M.tb* culture filtrate) in 25 μl of PBS was administered intranasally (19, 20).

### *M. tuberculosis* Infection and CFU Assay

*M.tb* H37Rv bacilli were grown in Middlebrook 7H9 broth supplemented with OADC and stored at −70°C until use. Infective inoculum of *M.tb* was prepared in PBS at a dose of 1 × 10^4^ bacteria and delivered intranasally to animals (19, 20). At specified time points post-challenge, animals were sacrificed and serial dilution of lung homogenates was plated in triplicate onto Middlebrook 7H10 plates and incubated at 37°C until ready for determination of the colony-forming units (CFU).

### Intravascular Staining to Discriminate Lung Parenchymal and Lung Vascular T Cell Populations

Intravascular immunostaining was carried out as previously described by us and others (15, 30). Briefly, monoclonal anti-CD45.2-Alexa Fluor 700 mAb (clone 104) (BD Pharmingen, San Jose, CA, USA) was prepared at 1 μg/250 μl concentration and injected intravenously via the tail vein. Within 3 min after injection, animals were sacrificed and bled. Blood was collected in heparin containing microcentrifuge tubes (40 units/ml) (Sigma-Aldrich, St. Louis, MO, USA) and kept at room temperature. Lung was removed with the trachea intact to perform bronchoalveolar lavage (BAL) and obtain BAL fluids. After BAL retrieval, lungs were collected in Hank's media. Spleen and lymph nodes were collected in RPMI 1640 medium. All the organs were kept in the dark on ice until further processing.

### Bronchoalveolar Lavage, Lung, Blood, Spleen, and Lymph Node Mononuclear Cell Isolation

In some experiments, the conventional BAL fluids were stored at −20°C for cytokine analysis. BAL cells were resuspended in complete RPMI medium supplemented with 10% fetal bovine serum, 1% penicillin—streptomycin, and 1% l-glutamine (cRPMI). Lung mononuclear cells were isolated after digestion with collagenase type 1 and ACK lysis of red blood cells as previously described (15, 19). Heparinized blood samples were treated twice with ACK lysis buffer (Invitrogen, Burlington, ON, Canada) to remove all red blood cells and washed with PBS. A single cell suspension of lymph nodes and spleens was made by crushing the organs using frosted glass slides. For spleen mononuclear cell isolation, red blood cells were lysed using ACK lysis buffer. All isolated cells were resuspended in cRPMI.

### T Cell Purification for *in vivo* Adoptive Transfer

In some experiments, CD8 T cells were purified from the single-cell suspension of lung tissue using a mouse CD8 T cell negative selection kit (STEMCELL Technologies, Vancouver, BC, Canada) according to the manufacturer's instructions. Purity (>90%) was confirmed by flow cytometry on Fortessa using FACSDiva Software (BD Biosciences). Purified cells were resuspended in PBS for adoptive transfer via the tail vein.

### Cell Stimulation, Tetramer Staining, Intracellular Cytokine Staining, and Flow Cytometry

The isolated mononuclear cells were seeded in a U-bottom 96-well plate at a concentration of 5 million cells/ml for BAL; 20 million cells/ml for lungs, spleens, and lymph nodes; and 10 million cells/ml for blood. For intracellular cytokine staining, mononuclear cells were incubated at 37°C in the presence of Golgi plug (5 mg/ml brefeldin A; BD Pharmingen, San Jose, CA, USA) with or without stimulation with a *M.tb* Ag85A-specific CD4 (LTSELPGWLQANRHVKPTGS) or CD8 (MPVGGQSSF) T cell peptide at a concentration of 1 μg/well for 5–6 h (19). Incubation was followed by washing, LIVE/DEAD Fixable Aqua Staining in PBS (Invitrogen, Burlington, ON, Canada), and blocking using CD16/CD32 Fc block mAb (clone 2.4G2) (1:150) (BD Pharmingen, San Jose, CA, USA) in 0.5% bovine serum albumin/PBS for 15 min on ice. Cells were then washed and stained using cell surface mAbs. After that, cells were washed, permeabilized, and stained for intracellular cytokines. In some experiments after Fc blockade, Ag85A-specific T cells were identified using a tetramer specific for the Ag85A CD8 T cell peptide (MPVGGQSSF) bound to BALB/c MHC class I allele H-2L^d^ (National Institutes of Health Tetramer Core, Atlanta, GA, USA) (19, 29). Cells were incubated for 1 h in the dark at RT with tetramers conjugated to PE fluorochrome and then stained for surface markers. Cells were then fixed using 1% paraformaldehyde/PBS at RT for 10–15 min and stored at 4°C until analysis using a flow cytometer. The mAbs used include Thy1.2-APC (clone 53-2.1), Thy1.1-PerCP-Cy5.5 (clone OX-7), CD3-PerCP-Cy5.5 (clone 145-2C11) (1:200), CD3-V450 (clone 17A2) (1:200), CD8a-PE-Cy7 (clone 53-6.7) (1:400), CD4-APC-Cy7 (clone RM4-5) (1:400), CD49a (α1 domain of VLA-1)-Alexa Fluor 647 (clone Ha31/8) (1:100), CD103-Biotin (clone 2E7) (1:100), Qdot-800-Streptavidin (1:500), IFN-γ-PerCP-Cy5.5 (clone XMG1.2) (1:200), CD69-BV605 (clone H1.2F3) (1:100), CD44-BV650 (clone IM7) (1:100), and CD62L-PerCP-Cy5.5 (clone MEL-14) (1:300) (Biolegend, Minneapolis, MN, USA). All mAbs were bought from BD Biosciences. Stained cells were run on Fortessa (BD Biosciences) and analyzed using FlowJo version 10 software (Tree Star, Ashland, OR, USA).

### T Cell Viability and Proliferation Assay

Viability of Ag85A-specific CD8 T cells in the lung was assessed by evaluating apoptotic and necrotic cells. Apoptotic cells were detected by annexin V staining with the apoptosis detection kit (Invitrogen, Burlington, ON, Canada). Briefly, after the staining with tetramer and surface mAbs, cells were stained with 5 μl of annexin V-FITC/APC (Invitrogen, Burlington, ON, Canada) according to the manufacturer's instruction. LIVE/DEAD Fixable Aqua Staining in PBS (Invitrogen, Burlington, ON, Canada) was performed to detect live cells. Cell proliferation was measured by monitoring BrdU incorporation into newly synthesized DNA as we described previously (24, 31). Briefly, 500 μg of BrdU was administered intranasally for a specified time period and cells incorporating BrdU were detected using mAb against BrdU according to the manufacturer's instruction (BD Biosciences, San Jose, CA, USA).

### *In vivo* CD4 T Cell Depletion

In some experiments, CD4 T cells were depleted using anti-CD4 mAb (clone GK1.5) (ATCC TIB-207; ATCC, Manassas, Virginia, USA) as previously described (24). Mice were inoculated intraperitoneally (i.p.) with 200 μg of anti-CD4 mAb and again 1 day after with 100 μg of anti-CD4 mAb. Thereafter, weekly administration of 100 μg of anti-CD4 mAb was done until the end of the experiment to maintain the state of CD4 T cell depletion.

### *In vivo* TNFα Neutralization

In some experiments, mice were inoculated i.n. every other day with 100 μg of anti-TNFα neutralizing mAb (clone XT3.11; Bio-X-Cell) until the end of experiment (32).

### Enzyme-Linked Immunosorbent Assay (ELISA) for Cytokine Measurement

Cytokine protein levels including interferon-γ (IFN-γ) and tumor necrosis factor-α (TNF-α) in BAL were measured using the respective DuoSet kit (R&D Systems, Burlington, ON, Canada).

### Statistical Analysis

All data were analyzed using GraphPad Prism software (version 6) (San Diego, CA, USA). For analysis of protection data, the difference was considered significant when *P* < 0.1 using a two-tailed Student *t* test for pairwise comparison with a confidence interval 90%. For all other data, the differences considered statistically significant were indicated as ^*^P < 0.05, ^**^P < 0.01, ^***^P < 0.001, and ^****^P < 0.0001 and a two-tailed Student *t* test was used for pairwise comparisons.

## Results

### Lung Recruitment and Induction of Ag-Specific CD8 T_RM_ Cells by RM-Pull Strategies With Pro-Inflammatory Agonists During the Effector Phase of T Cell Responses to Parenteral Immunization

**Figure 1:**
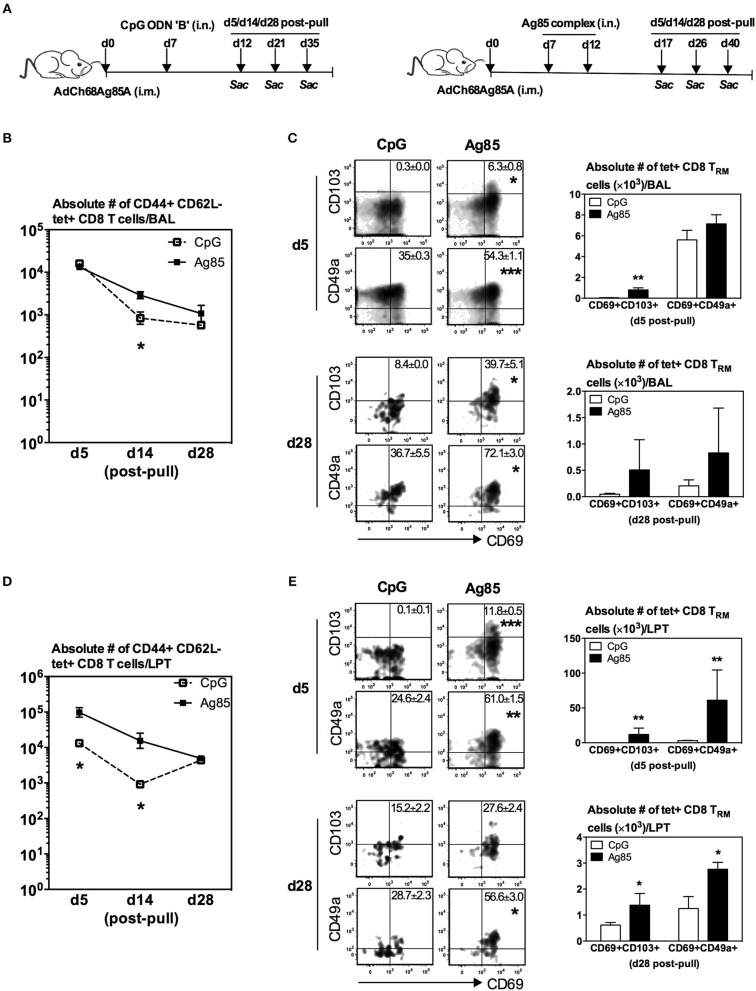
Lung recruitment and induction of Ag-specific CD8 T_RM_ by respiratory mucosal (RM)-pull with pro-inflammatory agonists during the effector phase of T cell responses to parenteral immunization. **(A)** Experimental schema. Mice were immunized parenterally (i.m.) with AdCh68Ag85A. At day 7 post-immunization, mice were administered via RM route (i.n.) with CpG (single dose) or Ag85 complex (two doses 5 days apart). Mice were then scarified at specified time points (day 5, day 14, and day 28) post-RM-pull, and mononuclear cells from BAL fluids and lungs were subjected to Ag85A tetramer and CD8 T_RM_ surface marker immunostaining. **(B)** Line graphs showing kinetic changes in numbers of tetramer+ cells in the airway. **(C)** Representative dotplots showing frequencies of tetramer+ CD8 T cells co-expressing CD69 and CD103, or CD69 and CD49a, and bar graphs showing numbers of tetramer+ CD8 T cells co-expressing CD69 and CD103, or CD69 and CD49a in the airway at day 5 and day 28 post-RM-pull. **(D)** Line graphs showing kinetic changes in numbers of tetramer+ cells in the lung parenchymal tissue (LPT). **(E)** Representative dotplots showing frequencies of tetramer+ CD8 T cells co-expressing CD69 and CD103, or CD69 and CD49a, and bar graphs showing numbers of tetramer+ CD8 T cells co-expressing CD69 and CD103, or CD69 and CD49a in the LPT at day 5 and day 28 post-RM-pull. Data are expressed as the mean ± S.E.M. of three mice/group/time point, representative of two independent experiments. *P < 0.05, **P < 0.01, ***P < 0.001 compared with CpG group.

It is known that parenteral-prime immunization alone is unable to induce bona fide *M.tb* Ag-specific T cell responses in the lung but Ag-independent and -dependent RM-pull strategies can be employed to recruit systemically activated Ag-specific CD8 T cells to the lung (14, 19, 20, 22). However, it remains unclear whether such parenteral prime-RM-pull strategies can all or differentially induce Ag-specific T_RM_. To address this question, we used a murine model of parenteral (intramuscular) prime immunization with a chimpanzee adenovirus-vectored TB vaccine bioengineered to express *M.tb* Ag85A (AdChAg85A) which is primarily a CD8 T cell activator (29). This was followed, at day 7 (the effector phase) post-parenteral priming, by RM-pull via intranasal delivery of an Ag-independent pro-inflammatory agonist (CpG) or a soluble *M.tb* Ag85 complex preparation (Figure 1A). Ag85A tetramer (Ag)-specific CD8 T cells (CD44+CD62L-tet+) and their T_RM_ phenotype (co-expression of CD103 and CD69 or CD49a and CD69) (16, 27) were examined in the airway (BAL) and lung parenchymal tissue (LPT). The T cells located in LPT and those in lung vasculature (LV) were differentiated by intravascular staining throughout this study (15, 30) (Supplementary Figure 1).

We found that numbers of total Ag-specific (tet+) CD8 T cells in the airway were by and large comparable between CpG and Ag85 groups at various time points post-RM-pull (Figure 1B). However, frequencies (dotplots) or numbers of Ag-specific T_RM_ CD8 cells (tet+CD69+CD103+ or tet+CD69+CD49a+) were greater in the airway at day 5 and day 28 in animals RM-pulled with Ag85 proteins than with CpG (Figure 1C). In comparison, numbers of total Ag-specific (tet+) T cells in the LPT compartment were significantly higher in Ag85-RM-pulled animals than in the CpG group up to 14 days post-RM-pull (Figure 1D). Furthermore, frequencies (dotplots) or numbers of Ag-specific CD8 T_RM_ cells (tet+CD69+CD103+ or tet+CD69+CD49a+) were markedly greater at day 5 and day 28 in the LPT of animals RM-pulled with Ag85 than in those with the CpG group (Figure 1E). Of note, the LPT of CpG-pulled animals had few CD8 T_RM_ cells at day 5 post-RM-pull (Figure 1E).

To examine whether such Ag85-specific Ag-determined differential induction of CD8 T_RM_ phenotype also occurred in non-Ag85A-specific T cells recruited into the lung in response to RM-pull, we analyzed the phenotype of non-Ag-specific CD8 T cells. While comparable numbers of non-Ag85A-specific CD8 T cells were recruited and retained in the lung following RM-pull with CpG or Ag85 proteins, only negligible frequencies of these cells assumed a T_RM_ phenotype (Supplementary Figures 2A–D). Taken together, the above data suggest that when carried out during the effector phase of parenteral TB immunization, RM-pull strategies with both Ag-independent and Ag-dependent proinflammatory agonists could recruit parenteral vaccine-primed Ag-specific T cells into the airway and LPT. However, specific cognate Ag has a greater effect on induction of CD8 T_RM_ phenotype in the lung.

### Induction of Ag-Specific CD8 T_RM_ Cells in the Lung by RM-Pull Strategy With Cognate Ag, but Not CpG, During the Memory Phase of T Cell Responses to Parenteral Immunization

**Figure 2:**
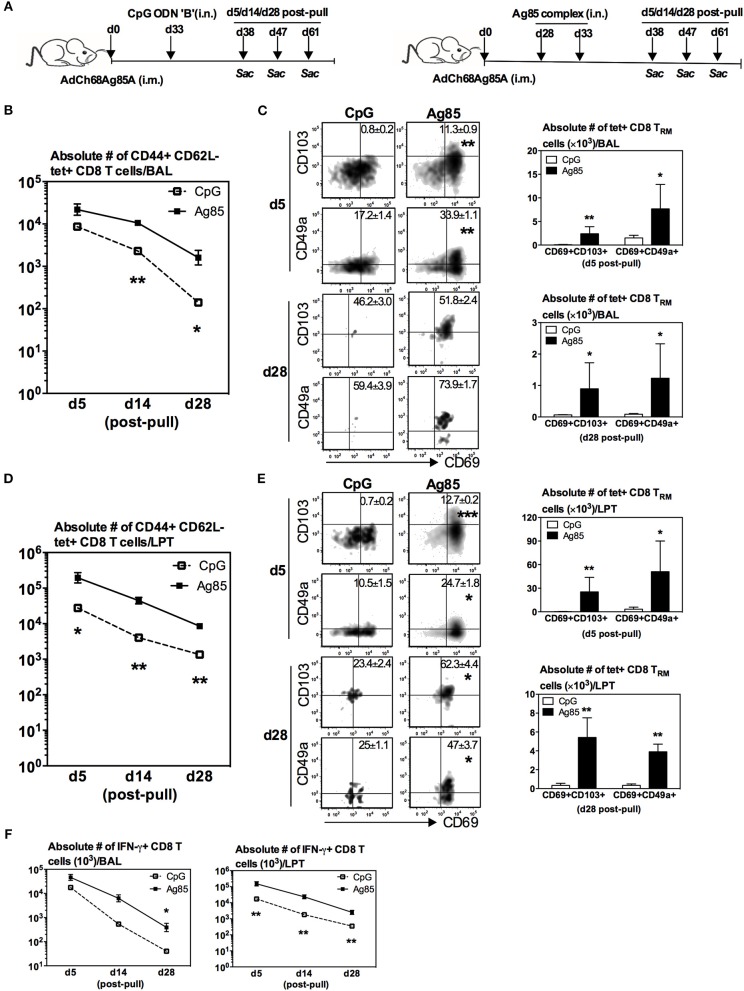
Induction of Ag-specific CD8 T_RM_ by cognate Ag-, but not by CpG-based RM-pull strategy during the memory phase of T cell responses to parenteral immunization. **(A)** Experimental schema. Mice were immunized i.m. with AdCh68Ag85A. At day 28 post-immunization, mice were administered i.n. with CpG (single dose) or Ag85 complex (two doses 5 days apart). Mice were then scarified at specified time points (day 5, day 14, day 28) post-RM-pull and mononuclear cells from BAL fluids and lungs were subjected to Ag85A tetramer and CD8 T_RM_ surface marker or intracellular cytokine immunostaining. **(B)** Line graphs showing kinetic changes in numbers of tetramer+ cells in the airway. **(C)** Representative dotplots showing frequencies of tetramer+ CD8 T cells co-expressing CD69 and CD103, or CD69 and CD49a, and bar graphs showing numbers of tetramer+ CD8 T cells co-expressing CD69 and CD103, or CD69 and CD49a in the airway at day 5 and day 28 post-RM-pull. **(D)** Line graphs showing kinetic changes in numbers of tetramer+ cells in the lung parenchymal tissue (LPT). **(E)** Representative dotplots showing frequencies of tetramer+ CD8 T cells co-expressing CD69 and CD103, or CD69 and CD49a, and bar graphs showing numbers of tetramer+ CD8 T cells co-expressing CD69 and CD103, or CD69 and CD49a in LPT at day 5 and day 28 post-RM-pull. (**F**) Absolute numbers of interferon-γ (IFN-γ)-producing CD8 T cells in the airway and LPT. Data are expressed as the mean ± S.E.M. of three mice/group/time point, representative of two independent experiments. *P< 0.05, **P < 0.01, ***P < 0.001 compared with CpG group.

While we have shown above that RM-pull during the effector phase of T cell activation with a type of pro-inflammatory agonist containing the cognate Ag, but not with CpG, is able to not only recruit parenteral vaccine-primed T cells but also promote their acquisition of T_RM_ phenotype in the lung, the timing of RM-pull is not practical for real-world applications. Furthermore, it would also be relevant to understand whether the effector and memory T cells recruited into the lung have differential capabilities to become T_RM_ cells. Hence, we next determined whether such strategy could still be effective when applied during the memory phase of T cell responses to parenteral immunization. To this end, using the model described in Figure 1A, RM-pull was performed with CpG or Ag85 complex proteins at day 28 (the memory phase) post-parenteral immunization. T cell responses in the lung were examined at day 5, day 14, and day 28 post-RM-pull (Figure 2A), and bona fide LTP T cells were determined via lung intravascular immunostaining (Supplementary Figure 3). RM-pull with CpG or Ag85 complex elicited comparable numbers of Ag-specific (tet+) CD8 T cells to the airway at day 5 post-RM-pull (Figure 2B). However, of note, the size of T cell population induced by CpG contracted much more quickly overtime than those by Ag85 complex (Figure 2B). Of importance, only the cells induced in the airway by cognate Ag85 Ag acquired a significant T_RM_ phenotype (Figure 2C, day 28 bar graph; tet+CD69+CD103+ or tet+CD69+CD49a+)_._ Thus, by day 28, few Ag-specific CD8 T cells were detected (Figure 2C, dotplots) with the absence of CD8 T_RM_ in the airway (Figure 2C) following CpG-based RM-pull. Similarly, Ag85 complex-based RM-pull resulted in significantly greater numbers of total Ag-specific (tet+) CD8 T cells between day 5 and day 28 in the LPT compartment (Figure 2D). Markedly increased frequencies and numbers of Ag-specific CD8 T_RM_ cells (tet+CD69+CD103+ or tet+CD69+CD49a+ T cells) were detected only in the LPT compartment of Ag85 complex-RM-pulled hosts, but not in that of CpG-pulled counterparts (Figure 2E). We also examined the functional quality of these Ag-specific CD8 T cells RM-pulled into the lung by assessing their cytokine production capacity in response to Ag stimulation. In keeping with kinetic changes in overall Ag-specific tet+ CD8 T cells in the BAL (Figure 2B) and LPT (Figure 2D), numbers of IFNγ-producing CD8 T cells were significantly greater at various time points both in the airway and LPT of Ag85 complex-RM-pulled hosts than in CpG-pulled counterparts (Figure 2F).

Thus, upon comparison with the data on RM-pull carried out during the effector phase of T cell responses (Figures 1A–E), apparently CpG-based RM-pull during the memory phase was unable to induce CD8 T_RM_ cells in the lung, in contrast to the potent effects by Ag85 complex-based RM-pull strategy (Figures 2A–E). Furthermore, Ag85 complex-mediated RM-pull carried out in the memory phase (day 28) seemed to have a greater effect on CD8 T_RM_ induction than when it was done in the effector phase (day 7) (Table 1).

**Table 1:** Absolute # of tet+CD69+CD103+ CD8 T_RM_ cells (10^3^) at day 28 post pull.

	**Effector**	**Memory**
BAL	0.5 ± 0.2	0.9 ± 0.3
LPT	1.4 ± 0.2	5.4 ± 0.7^*^

*Comparison of numbers of tet+CD69+CD103+ CD8 T_RM_ cells (10^3^) in BAL and LPT at day 28 post-cognate Ag-based RM-pull carried out in the effector and memory phases of T cell responses to parenteral immunization. Following RM-pull with Ag85 complex during the effector and memory phase of T cell responses, mice were sacrificed at 28 days after RM-pull. Table shows numbers of tetramer+ Ag-specific CD8 T cells co-expressing CD69 and CD103 in BAL and the lung parenchymal tissue (LPT). Data are presented as the mean ± S.E.M. of three mice/group, representative of two independent experiments*.

* *P < 0.05*.

To address whether the striking induction of Ag-specific CD8 T_RM_ phenotype by Ag85-based RM-pull strategy was primarily a local immune event within the lung mucosal environment, we examined and compared T cells within the LV following CpG or Ag85 RM-pull. Of interest, numbers of total Ag-specific (tet+) CD8 T cells in the LV were very similar between CpG and Ag85 groups at all time points (Supplementary Figure 4A), in contrast to a marked difference in such T cells seen in the lung (Figures 2B,D). Furthermore, different from its potent effects in the lung, Ag85 RM-pull did not lead to induction of expression of T_RM_ -associated surface markers, hence the absence of Ag-specific CD8 T_RM_ in the LV of both CpG and Ag85 RM-pulled animals (Supplementary Figure 4B). The above data together suggest that when RM-pull strategy was carried out during the memory phase of T cell responses following parenteral immunization, the Ag-independent approach using CpG is unable to induce Ag-specific CD8 T_RM_ cells in the airway and LPT. In contrast, the Ag-dependent approach using cognate antigens (Ag85 complex proteins) is particularly potent in inducing such T_RM_ cells upon T cell entry to the lung.

**Figure 3:**
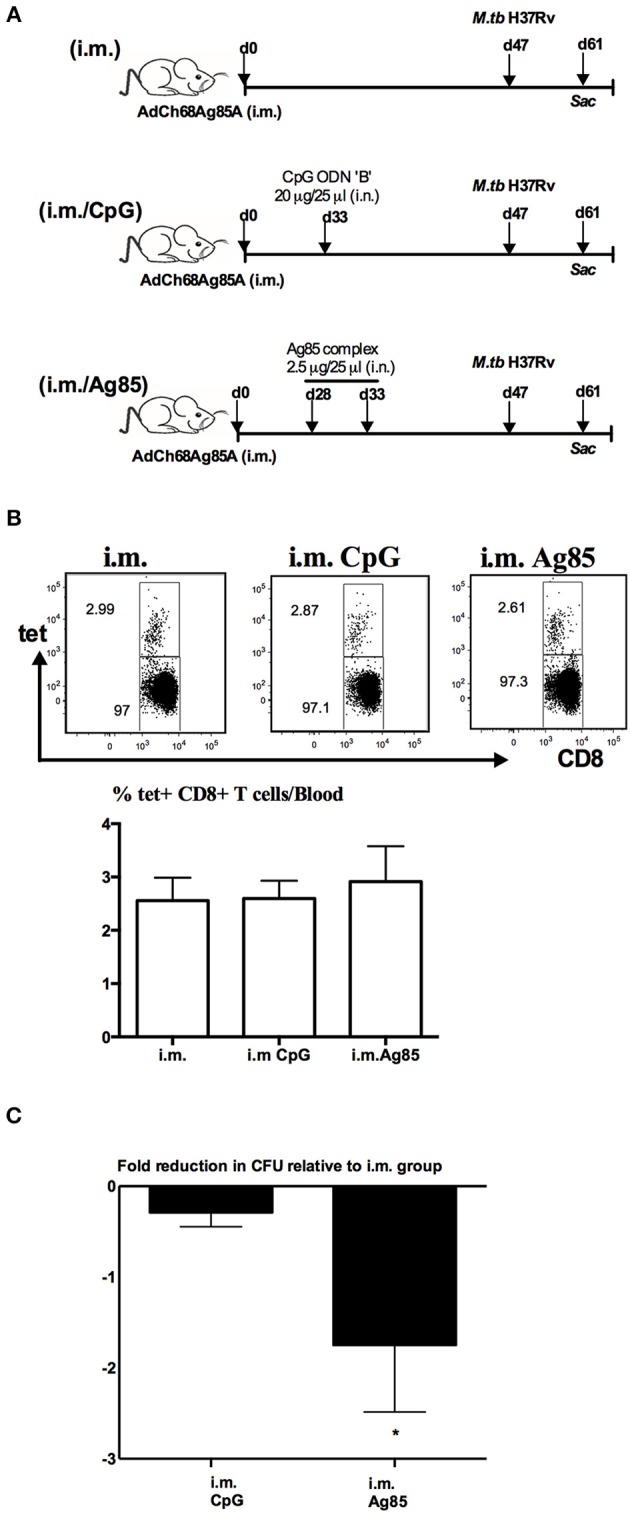
Enhanced control of mycobacterial infection in the lung by cognate Ag-based RM-pull strategy in parenteral vaccine-primed animals. **(A)** Experimental schema. Mice were left untreated, only immunized i.m. with AdCh68Ag85A (i.m.), or immunized i.m. and received i.n. delivery of CpG (single dose) (i.m. CpG) or Ag85 complex proteins (two doses 5 days apart) (i.m. Ag85) at day 33 or day 28 post-immunization. Some mice were sacrificed before infection for T cell analysis and other groups of mice were infected with M.tb H37Rv and the lungs were harvested for analysis 2 weeks after infection. **(B)** Representative dotplots and bar graph comparing frequencies of circulating Ag85A-specific CD8 T cells in i.m., i.m./CpG, and i.m./Ag85 groups **(C)** Bar graph showing changes in the level of *M.tb* infection in the lung of i.m. CpG and i.m. Ag85 groups. Data are expressed as the mean ± S.E.M of fold changes relative to i.m. group from 10 mice/group of two independent experiments. *P < 0.05 compared with CpG group.

We next determined whether relative levels of RM-pull-induced Ag-specific T_RM_ cells might be associated with different levels of anti-TB protective immunity in the lung. To this end, mice were parenteral AdChAg85A vaccine-primed and RM-pulled in the memory phase of immune responses without or with either CpG or Ag85 complex proteins, and subsequently infected with *M.tb* 14 days after RM-pull and lung infection levels assessed at day 14 post-infection (Figure 3A). Right before infection, the three groups of mice had comparable frequencies of Ag-specific CD8 T cells in the circulation (Figure 3B). Post-infection and consistent with the previous studies (7, 15, 19, 20), we did not observe any improved protection by parenteral vaccination alone (i.m. group) when compared with naïve animals (around 4.5 log CFU). As expected, RM-pull with CpG (i.m. CpG group) only marginally improved bacterial control relative to that in the i.m. group (Figure 3C), in keeping with its weak ability to induce T_RM_ cells in the lung during the memory phase of T cell responses to parenteral priming (Figure 2). In contrast, RM-pull with Ag85 proteins (i.m. Ag85 group) led to significantly improved bacterial control in the lung (Figure 3C), suggesting that increased T_RM_ cells induced by cognate Ag-dependent RM-pull strategy in parenteral TB vaccine-primed hosts are associated with improved lung protection.

### Induction of Ag-Specific CD4 T_RM_ Cells by RM-Pull Strategy With Cognate Ag, but Not by CpG-Mediated, During the Memory Phase of T Cell Responses to Parenteral Immunization

**Figure 4:**
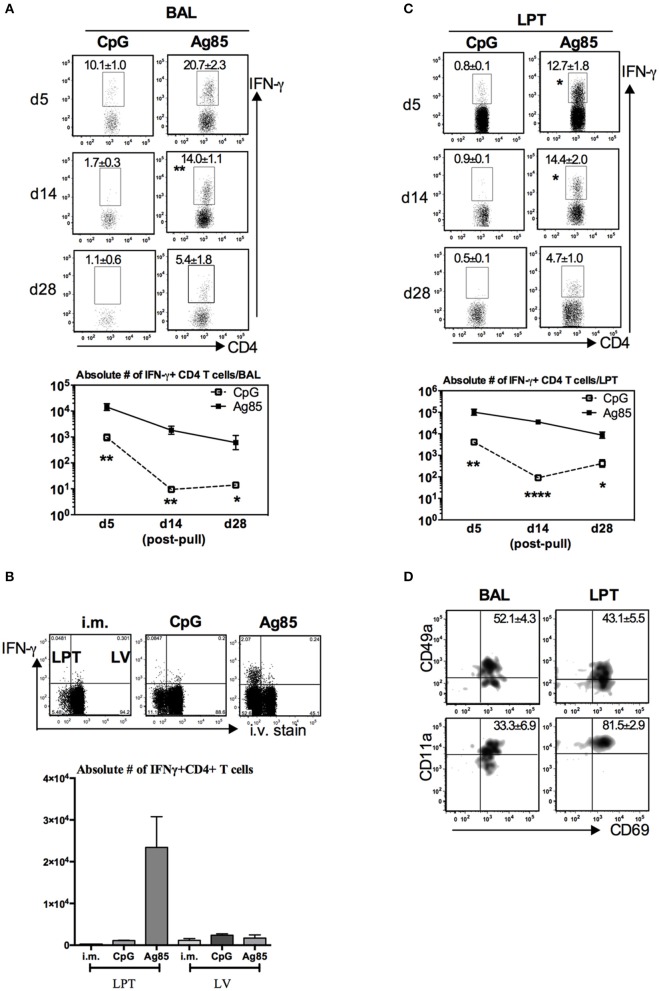
Induction of Ag-specific CD4 T_RM_ cells by cognate Ag-based but not by CpG-based RM-pull strategy during the memory phase of T cell responses to parenteral immunization. Mice were immunized parenterally (i.m.) with AdCh68Ag85A. At day 28 post-immunization, mice were administered i.n. with CpG (single dose) or Ag85 complex (two doses 5 days apart) as described in Figure 2A. Mice were then scarified at specified time points (day 5, day 14, and day 28) post-RM-pull, and mononuclear cells from BAL fluids and lungs were subjected to *ex vivo* stimulation with Ag85A CD4 peptides, CD4 T_RM_ cell surface marker, or intracellular cytokine immunostaining. **(A)** Representative dotplots showing frequencies of CD4+IFN-γ+ cells in the airway (BAL). Line graphs showing kinetic changes in numbers of IFN-γ+CD4 T cells in the airway post-RM-pull. **(B)** Representative dotplots comparing distribution of CD4+IFN-γ+ cells in the lung parenchymal tissue (LPT) and the lung vasculature (LV) of i.m., i.m./CpG, and i.m./Ag85 mice. Bar graph showing absolute number of CD4+IFN-γ+ cells in LPT and LV. **(C)** Representative dotplots showing frequencies of CD4+IFN-γ+ cells in the LPT. Line graphs showing kinetic changes in numbers of IFN-γ+CD4 T cells in the LPT post-RM-pull. **(D)** Representative dotplots show frequencies of IFN-γ+CD4+ T cells co-expressing CD69 and CD49a, or CD69 and CD11a in the airway and LPT at day 28 post-RM-pull. Data are expressed as the mean ± S.E.M. of three mice/group/time point, representative of two independent experiments. *P < 0.05, **P < 0.01, ****P < 0.0001 compared with CpG group.

A recent study suggests that CD4 T cells are involved in CD8 T_RM_ development in the lung following influenza infection (33). Given that thus far, we have seen the cognate Ag-dependent, but not the Ag-independent, RM-pull strategy to potently induce Ag-specific CD8 T_RM_ in the lung, we next investigated the potential mechanisms underlying such difference. We first examined whether CD4 T cells were induced differentially by these two RM-pull strategies. Using the experimental setup described in Figure 2A, we examined Ag-specific CD4 T cells in the airway and LPT by evaluating *ex vivo* CD4 T cell peptide-stimulated IFN-γ+ CD4 T cell responses in conjunction with their expression of CD4 T_RM_-associated surface markers CD69, CD49a, and CD11a (28). Consistent with its inferior ability to recruit and retain Ag-specific CD8 T cells in the lung (Figures 2B,D,F), CpG RM-pull-induced Ag-specific CD4 T cells disappeared quickly, and by day 14 and day 28 post-pull, they became almost undetectable in the airway and LPT (Figures 4A,C). Thus, CpG RM-pulled LPT was indistinguishable from the unpulled i.m. prime LPT (Figure 4B). In sharp contrast, RM-pull with Ag85 proteins induced a significantly higher magnitude of Ag-specific CD4 T cell responses in the airway and LPT at all time points (Figures 4A–C), and the majority of these CD4 T cells in the airway and LPT were of the CD4 T_RM_ phenotype, being all positive for CD69 and some for CD49a and CD69 (Figure 4D). As was the case for CD8 T_RM_ differentiation, induction of CD4 T_RM_ cells by Ag85-based RM-pull occurred locally in the lung as Ag-specific CD4 T cells within the LV did not express any of CD4 T_RM_-associated surface molecules (Supplementary Figure 5). These data suggest that consistent with its potent effects on induction of CD8 T_RM_ cells in the lung, RM-pull with cognate Ag85 proteins also induces significant Ag-specific CD4 T_RM_ responses in parenteral vaccine-primed animals, whereas RM-pull with CpG lacks such effects.

### Induction of Ag-Specific CD8 T_RM_ Cells in the Lung by RM-Pull Strategy Requires CD4 T Cells

**Figure 5:**
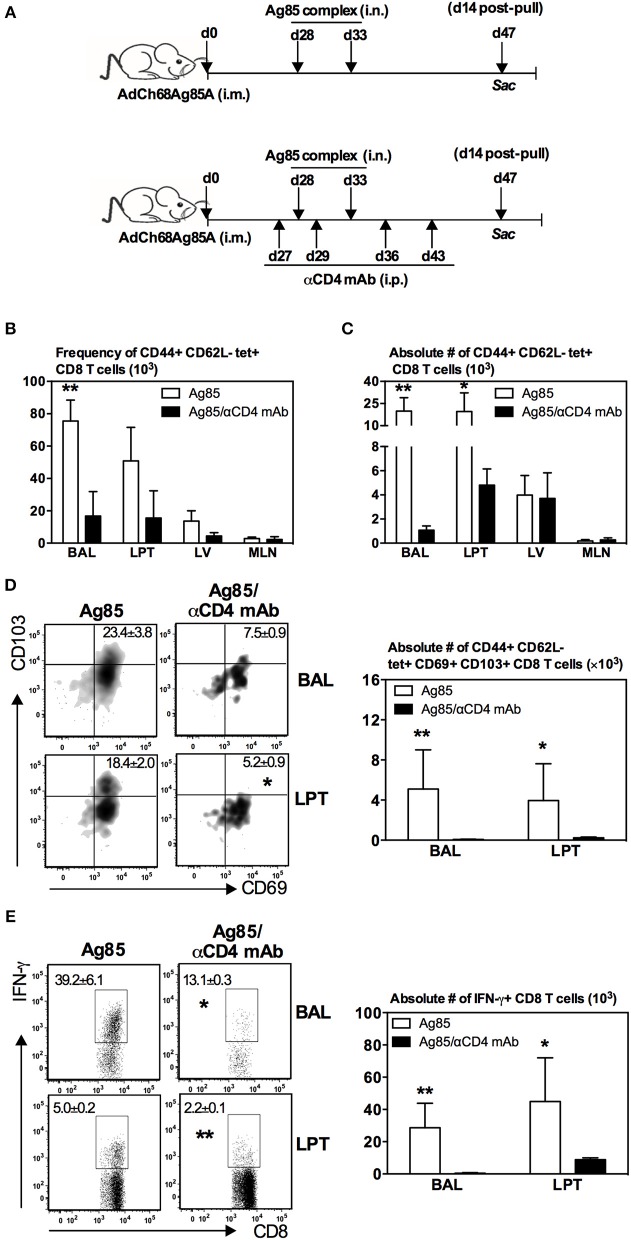
Induction of Ag-specific CD8 T_RM_ cells in the lung by RM-pull strategy requires CD4 T cells. **(A)** Experimental schema. Mice were immunized i.m. with AdCh68Ag85A. At day 28 post-immunization, mice were administered i.n. with Ag85 complex proteins (two doses 5 days apart). In a group of mice (Ag85/αCD4 mAb), CD4 T cells were depleted i.p. by injection of αCD4 mAb a day before the first dose of Ag85 complex and then a day after the last dose of Ag85 complex. Depletion of CD4 T cells was maintained by repeated αCD4 mAb injections every 7 days until the termination of experiment. Mice were sacrificed 14 days after the final Ag85 dose. Mononuclear cells from BAL and lungs were subjected to *ex vivo* stimulation with Ag85A CD8 peptides, and Ag85A tetramer and CD8 T_RM_ surface marker or intracellular cytokine immunostaining. Bar graphs showing frequencies **(B)** and absolute numbers **(C)** of tetramer+ cells in the airway (BAL), lung parenchymal tissue (LPT), lung vasculature (LV), and mediastinal lymph nodes (MLN). **(D)** Representative dotplots and bar graph showing frequencies and numbers of tetramer+ CD8 T cells co-expressing CD69 and CD103, respectively. **(E)** Representative dotplots and bar graph depicting frequencies and numbers of IFN-γ+ CD8 T cells. Data are presented as the mean ± S.E.M. of three mice/group/time point, representative of two independent experiments. *P < 0.05, **P < 0.01 compared with Ag85 group.

The correlation of CD4 T cell responses with induction of CD8 T_RM_ cells in the lung by cognate Ag-based RM-pull strategy (Figures 2, 4) suggested a mechanistic role for CD4 T cells in the development of CD8 T_RM_ cells. To investigate this further, parenteral vaccine-primed animals were depleted of CD4 T cells with a depleting mAb prior to RM-pull with Ag85 complex proteins and CD4 T cell depletion was maintained by repeated anti-CD4 T cell mAb injections until the termination of the experiment (day 14 post-second Ag85 i.n. delivery) (Figure 5A). This approach allowed proper parenteral priming of Ag-specific CD8 T cells in the presence of CD4 T cells and the focus to examine the role of CD4 T cells in RM-pull-induced CD8 T_RM_ cell responses. We found that both frequencies (Figure 5B) and numbers (Figure 5C) of Ag-specific (tet+) CD8 T cells were markedly reduced in the airway and LPT in CD4 T cell-depleted animals (Ag85/αCD4 mAb) compared to CD4 T cell-competent animals (Ag85). However, they remained comparable in the LV and mediastinal draining lymph nodes (MLN) (Figures 5B,C). Of note, the Ag-specific CD8 T cells recruited into the airway and LPT of CD4 T-cell-depleted animals failed to acquire a T_RM_ phenotype (CD69+CD103+) (Figure 5D). Besides the lack of expression of T_RM_ surface molecules, Ag-specific CD8 T cells in the airway and LPT in such animals also exhibited a much reduced ability to produce IFNγ upon Ag-restimulation (Figure 5E). Thus, the above data suggest that following RM-pull strategy with a cognate Ag in parenteral vaccine-primed animals, CD4 T cells are involved in the development of CD8 T_RM_ cells.

### CD4 T Cells Regulate Ag-Specific CD8 T_RM_ Responses via Their Local Effects on Proliferation/Survival, but Not Recruitment, of CD8 T Cells in the Lung

**Figure 6:**
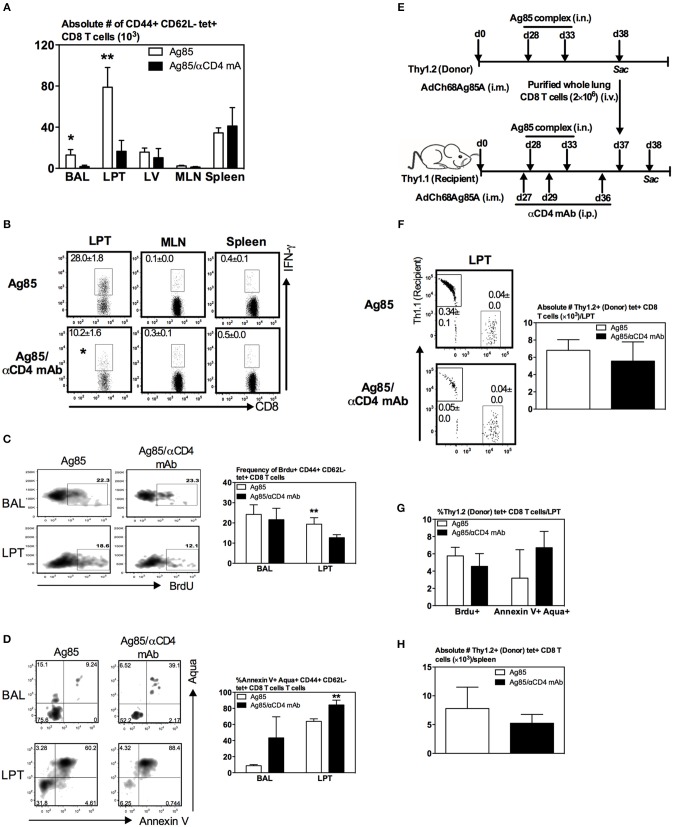
Regulation of Ag-specific CD8 T_RM_ responses by CD4 T cells via their effects on proliferation/survival, but not recruitment, of CD8 T cells in the lung. Mice were immunized i.m. with AdCh68Ag85A. At day 28 post-immunization, mice were administered i.n. with Ag85 complex (two doses 5 days apart) (Ag85 group). In a separate group of mice, CD4 T cells were depleted by i.p. injection of αCD4 mAb 1 day before and after RM-pull (Ag85/αCD4mAb group). BrdU was administered i.n. once daily for four consecutive days after the last dose of Ag85 complex proteins. Mice were sacrificed 5 days after the final administration of Ag85 complex, and mononuclear cells from BAL, lungs, MLN, and spleen were subjected to *ex vivo* stimulation with Ag85A CD8 peptide, and immunostaining for tetramer, BrdU, and Annexin V or intracellular cytokines. **(A)** Bar graph showing numbers of tetramer+ CD8 T cells in BAL, LPT, LV, MLN, and spleen. **(B)** Representative dotplots depicting frequencies of IFN-γ+ CD8 T cells. **(C,D)** Representative dotplots and bar graphs showing frequencies of BrdU+ **(C)** and Annexin V+Aqua+ **(D)** tetramer+ CD8 T cells out of total tetramer^+^ cells in BAL and LPT. **(E)** Experimental schema. Thy1.2 and Thy1.1 congenic mice were immunized i.m. with AdCh68Ag85A followed by RM-pull with Ag85 complex. CD8 T cells purified from Thy1.2 donor lungs were transferred i.v. into Th1.1 recipient mice post-Ag85 delivery and CD4 T cell depletion. BrdU was administered i.n. once after T cell transfer. Lung mononuclear cells were stained for tetramer, BrdU, and Annexin V 24 h post-T cell transfer. **(F)** Representative dotplots showing frequencies of Thy1.2 (donor) and Thy1.1 (recipient) and tetramer+ CD8 T cells out of total cells in LPT. Bar graph comparing numbers of Thy1.2 (donor) tetramer+ CD8 T cells in LPT between CD4 T cell-helped (Ag85) and -unhelped (Ag85/αCD4mAb) groups. **(G)** Bar graph comparing frequencies of BrdU+ or Annexin V+Aqua+ Thy1.2 (donor) tetramer+ CD8 T cells in LPT of CD4 T cell-helped (Ag85) and -unhelped (Ag85/αCD4mAb) groups. **(H)** Bar graph comparing numbers of Thy1.2 (donor) tetramer+ CD8 T cells in the spleen of CD4 T cell-helped (Ag85) and -unhelped (Ag85/αCD4mAb) groups. Data are presented as the mean ± S.E.M. of three mice/group, representative of two independent experiments. *P < 0.05, **P < 0.01 compared with Ag85 group.

So far, we have observed that numbers of both Ag-specific CD8 T cells and CD8 T_RM_ were seen markedly reduced in the lung of CD4 T cell-depleted animals (Figures 5B–D). To further examine the role of CD4 T cells in CD8 T_RM_ responses in the lung, we determined whether CD4 T cells were involved in the expansion of Ag-specific memory CD8 T cells in the secondary lymphoid organs. To this end, we employed a similar experimental approach in Figure 5A except that the animals were terminated at 5 days after the final delivery of Ag85 complex, instead of 14 days, and Ag-specific CD8 T cells were assessed in the lung, circulation, and secondary lymphoid organs. The choice of this 5-day time point allowed us to examine the peak immune responses in these tissue sites. We found that numbers of Ag-specific tet+ T cells were markedly reduced in the airway and LPT of CD4 T cell-depleted animals as early as 5 days post-RM-pull (Figure 6A). However, numbers of such T cells remained comparable in LV, draining lymph nodes (MLN), and spleen outside of the lung in both CD4 T cell-competent and -depleted animals (Figure 6A). A similar profile was also observed with Ag-stimulated IFNγ-producing CD8 T cells in these tissue sites (Figure 6B). These data suggest that CD4 T cells were not required for the expansion of peripheral Ag-specific memory CD8 T cells induced by parenteral vaccination.

The above findings (Figures 5B,C, 6A,B) together suggest a local role by CD4 T cells after the arrival of Ag-specific memory CD8 T cells at the lung. This prompted us to examine whether CD4 T cells played a role locally in the lung via modulating the proliferation and survival of Ag-specific CD8 T cells. To this end, we employed a BrdU incorporation assay in conjunction with annexin V and aqua staining. Mice were immunized and RM-pulled with Ag85 complex as described in Figure 5A. BrdU was administered i.n. once daily for four consecutive days after the last administration of cognate Ag and animals were sacrificed at day 5 post-last BrdU delivery (day 42 post-i.m.). Besides BrdU treatment, a group of mice were also depleted of CD4 T cells at time points as depicted in Figure 5A. The degree of BrdU incorporation, the expression of apoptotic marker annexin V, and the level of cell death among Ag-specific CD8 T cells in the airway and LPT were examined by FACS. We found that while the proliferating, BrdU+ Ag-specific tet+ CD8 T cells in the airway did not differ between CD4 T-cell-competent and -depleted animals (Figure 6C), frequencies of such proliferating T cells significantly decreased in the LPT of CD4 T-cell-depleted animals compared to those in CD4 T-cell-competent counterparts (Figure 6C). Furthermore, decreased T cell proliferation rates were accompanied by an increase in cell apoptosis/death of these cells in the airway and LPT of CD4 T-cell-deficient animals (Figure 6D). These data suggest that CD4 T cells contribute to CD8 T_RM_ development locally in the lung via regulating CD8 T cell proliferation and survival.

Besides regulation of CD8 T cell proliferation and survival by CD4 T cells, CD4 T cells might also be involved in CD8 T cell recruitment. In other words, much reduced CD8 T_RM_ cells in the lung following CD4 T cell depletion in Ag85 RM-pulled hosts could have been due in part to a non-conducive lung microenvironment for the recruitment of circulating Ag-specific CD8 T cells. To investigate if this could be the case, we employed a T cell adoptive transfer approach. Thus, congenic CD8 T cells (Thy1.2^+^CD45.2^+^) were purified from the lung of parenteral-primed and Ag85 RM-pulled animals and were transferred i.v. into Thy1.1^+^ recipient mice that were already parenteral-primed and RM-pulled with cognate Ag in the presence (Ag85) or absence (Ag85/αCD4 mAb) of CD4 T cells (Figure 6E). One day after T cell transfer, animals were sacrificed and their LPT was analyzed for the presence of Thy1.1^+^tet+ (recipient) and Thy1.2^+^ tet+ CD8 T cells (donor) among the total cells in the LPT. As expected from the earlier data (Figures 5, 6A,B), the frequency of endogenous (recipient) Thy1.1 tet+ T cells was significantly lower in the LPT of CD4 T-cell-depleted mice (Ag85/αCD4 mAb) than in the CD4 T-cell-competent control group (Ag85) (0.34 vs. 0.05%) (Figure 6F). In contrast, both the frequency of intravenously transferred donor CD8 T cells (Thy1.2^+^) and the number of donor Ag-specific CD8 T cells (Thy1.2+tet+) in the LPT were comparable between CD4 T-cell-competent (Ag85) and CD4 T-cell-depleted (Ag85/αCD4 mAb) groups (Figure 6F). These results were not influenced by cell proliferation/survival or geographical tissue distribution of the donor cells in these groups of animals as we found the donor Ag-specific CD8 T cells (Thy1.2+tet+) to demonstrate similar rates of proliferation, survival, and splenic distribution (Figures 6G,H). Together, the above data suggest that CD4 T cells play a role in regulating the levels of Ag-specific CD8 T_RM_ responses primarily via their local effects on T cell proliferation/survival, but not T cell recruitment in the lung.

### Involvement of TNF-α in Induction of CD8 T_RM_ Cells in the Lung by RM-Pull Strategy

**Figure 7:**
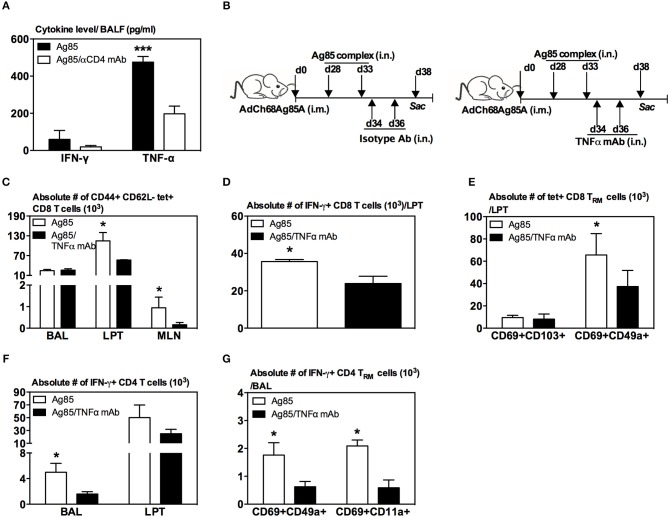
Involvement of TNF-α in induction of CD8 T_RM_ cells in the lung by RM-pull strategy. **(A)** Bar graph demonstrating levels of IFN-γ and TNF-α in BAL of i.m.-immunized animals at day 5 following RM-pull with Ag85 complex proteins without (Ag85) and with CD4 T cell depletion (Ag85/αCD4 mAb). **(B)** Experimental schema. Mice were immunized i.m. with AdCh68Ag85A. At day 28 post-immunization, mice were administered i.n. with Ag85 complex (two doses 5 days apart). The mice were depleted of TNF-α by i.n. delivery of an isotype Ab or TNF-α mAb every other day starting at day 1 post-RM-pull. At day 5 post-RM-pull, mononuclear cells from BAL, LPT, and MLN were *ex vivo* stimulated with Ag85A CD4 or CD8 peptides, and immunostained for tetramer, T_RM_ surface markers, and intracellular cytokines. **(C)** Bar graph demonstrating numbers of tetramer+ CD8 T cells in BAL, LPT, and MLN. **(D)** Bar graph showing numbers of IFN-γ+ CD8 T cells in LPT. **(E)** Bar graph showing numbers of tetramer+ CD8 T cells co-expressing CD69 and CD103, or CD69 and CD49a in LPT. **(F)** Bar graph showing numbers of IFN-γ+ CD4 T cells in BAL and LPT. **(G)** Bar graph showing numbers of IFN-γ+ CD4 T cells co-expressing CD69 and CD49a, or CD69 and CD11a in BAL. Data are presented as the mean ± S.E.M. of three mice/group, representative of two independent experiments. *P < 0.05, ***P < 0.001 compared with Ag85A group.

Since recent influenza studies have implicated certain cytokines such as CD4 T-cell-derived IFN-γ and TNF-α in lung CD8 T_RM_ cell responses (33), we first examined whether IFN-γ and TNF-α were differentially produced in the lung of CD4 T-cell-competent and -depleted animals following parenteral-primed and RM-pull treatment protocol. At 5 days post-RM-pull with cognate Ag (Ag85), the level of TNF-α, but not IFN-γ, markedly increased in the lung of CD4 T-cell-competent animals whereas deficiency of CD4 T cells (Ag85/αCD4 mAb) led to a significant reduction of TNF-α production in the lung (Figure 7A). These data thus suggested a role of CD4 T-cell-derived TNF-α in inducing CD8 T_RM_ cells in our model.

To further investigate the role of TNF-α, mice were parenteral-primed and RM-pulled with Ag85 complex proteins at 28 days post-parenteral priming (Figure 7B). In another group, TNF-α was neutralized locally in the lung by two repeated intranasal deliveries of anti-TNF antibodies after the final delivery of Ag85 (Figure 7B). Repeated Ag85 protein deliveries before TNF neutralization would allow the establishment of Ag-specific CD8 T cell responses in the lung as we previously found that a single Ag85 complex delivery was not able to induce Ag-specific CD8 T cell responses in the lung (20). Animals were sacrificed 5 days after the final delivery of Ag85 complex. We found that TNF-α neutralization (Ag85/TNFα mAb) led to significantly reduced Ag-specific CD8 T cells of effector memory phenotype (CD44+CD62L-tet+) in the LPT and MLN, but not in the airway, compared to the control (Ag85) (Figure 7C). Reduction of these Ag-specific CD8 T cells in the LPT was accompanied by significantly reduced IFN-γ-producing CD8 T cells in the LPT of TNF-α-neutralized animals (Figure 7D). Of importance, phenotypic conversion of Ag-specific CD8 T cells to CD69+CD49a+ CD8 T_RM_ cells was also impaired in the LPT of TNF-α-neutralized hosts (Figure 7E). Furthermore, lack of TNF-α also led to significantly reduced Ag-specific CD4 T cells (Figure 7F) and CD4 T_RM_ responses (Figure 7G). These data together suggest TNF-α to be involved in the induction of CD8 T_RM_ cells in the lung in our model.

## Discussion

It is now widely recognized that the non-circulating T_RM_ cells at the mucosa are critical to host defense (26–28). Thus, effective immunization strategies are expected to generate T_RM_ cells at the mucosal entry site of pathogens such as *M.tb* (4). Since the airways and lung parenchyma like other mucosal tissue sites (34) are highly restricted for T cell entry (15), the parenteral route of immunization as the most commonly used immunization strategy is unfortunately ineffective in inducing bona fide RM-associated T_RM_ cells (15–19). To this end, various RM-pull strategies have been explored to mobilize the circulating T cells into the lung in parenteral TB vaccine-primed hosts. These strategies include RM delivery of Ag-independent chemokines, TLR ligands, and microbial agents or specific *M.tb* Ag preparations (14, 19, 20), and RM deliveries of *M.tb* Ags alone were shown incapable of inducing T cell immunity (19, 20). However, it has remained unclear whether Ag-independent and Ag-dependent RM-pull strategies differ in their ability to induce T_RM_ cell differentiation in the lung mucosa of parenteral TB vaccine-primed hosts. Investigation of this question is important to identifying the ways to effectively improve mucosal protective immunity of parenteral TB vaccine strategies. Furthermore, it will help us to understand whether frequent exposure of human respiratory system to environmental-borne inflammatory agents (35) may facilitate the development of Ag-specific T_RM_ cells in the lung, given the fact that almost all of the current human vaccines including BCG are parenterally administered. Here, we show that both Ag-independent pro-inflammatory (CpG) and Ag-dependent (Ag85A) RM-pull strategies could all mobilize parenteral vaccine-primed effector or effector memory T cells into the lung. However, the effective conversion to T_RM_ cells in the lung requires the use of a cognate Ag-dependent RM-pull strategy. Such T_RM_ cells in our study are defined as CD69+CD103+ or CD69+CD49a+ CD8 T cells. Growing evidence suggests the presence of heterogeneous T_RM_ cells at the same tissue site (26), which explains the non-matching sizes of these two populations seen in our study. Besides cognate antigens, we find that cognate Ag-elicited CD4 T cell help is also required for CD8 T_RM_ formation. We further show that fully differentiated memory T cells following parenteral prime immunization have a much greater potential to differentiate into T_RM_ cells in the lung than the effector T cells.

Our finding that both Ag-independent and Ag-dependent RM-pull strategies are similarly able to recruit into the lung of parenteral-primed T cells with an adenoviral-based TB vaccine suggests a role of pro-inflammatory signals triggered by innate immune-activating properties of these strategies. Indeed, we have previously shown the requirement of chemokines locally in the lung for T cell recruitment in a parenteral DNA-based TB vaccine model (20). However, whether the recruited Ag-specific T cells, particularly the Ag-specific memory T cells, will sustain and differentiate into T_RM_ cells is critically dependent on the local presence of the cognate Ag, Ag85, that is expressed by the vaccine used to parenterally prime the host in our study. We have provided the evidence that the cognate Ag-based RM-pull strategy promotes the differentiation of lung CD8 T_RM_ cells via stimulating the proliferation of Ag-specific CD8 T cells (31) and inducing CD4 T cells within the lung. The important role of specific Ags in driving CD8 T_RM_ cell differentiation in the lung has also recently been observed in a parenteral influenza infection and RM-pull model (21, 23). Although such models of productive flu infection differ very much from our parenteral TB vaccination model where the viral backbone is replication-deficient, the findings together indicate that the lung represents a unique mucosal immune environment in its requirement of specific Ags for T_RM_ cell differentiation. This illustrates an important difference in T_RM_ differentiation between the lung and other major mucosal sites since it has been shown that only the T cell recruitment, but not necessarily the specific Ag, is required for T_RM_ cell responses in the genital tract, skin, and intestine (34, 36, 37). The data from our current study suggest that RM-pull strategies merely aiming to create an inflamed microenvironment conducive to recruiting parenteral vaccine-primed T cells into the lung will not be effective enough in establishing sustained CD8 T_RM_ cells. Effective RM-pull strategies should include both pro-inflammatory property and specific Ags that are expressed by parenteral vaccine. Moreover, our findings also imply that repeated exposure to environmentally borne irrelevant agents of human respiratory tract will unlikely spur the generation of sustained Ag-specific T_RM_ cells on RM surfaces.

The CD4 T cell help has long been known to play an important role in the development of central/effector memory CD8 T cells (38). Here, we show that besides cognate Ags, cognate Ag-elicited CD4 T cell help is also critical to CD8 T_RM_ cell responses in the lung of parenteral TB vaccine-primed hosts. Indeed, we observed that while cognate Ag-based RM-pull strategy potently induced CD8 T_RM_ responses, which were accompanied by a striking CD4 T_RM_ cell response, removal of CD4 T cell help by means of CD4 T cell depletion resulted in impaired CD8 T_RM_ cell differentiation. We provide further evidence that CD4 T cells likely mediate CD8 T_RM_ cell development in the lung via their production of TNF-α, thus lending further support to the role of this cytokine in CD8 T_RM_ cell differentiation identified in other models (32, 39). CD4 T cell help was also shown to be required for CD8 T_RM_ cell development in the lung in a respiratory influenza model (39). Both this study and our current study indicate the lack of CD4 T cell help to only impact CD8 T_RM_ cell differentiation but not CD8 T cell recruitment in the lung. CD4 T_RM_ cells induced by respiratory viral-vectored TB vaccine was also found to be critical to immune protection (40). These together suggest the importance of consideration of CD4 T cells in selection of types of cognate Ags to be included in RM-pull strategies. In this regard, the use of whole protein-based cognate Ags capable of engaging both CD4 and CD8 T cells would be advantageous over cognate CD8 T cell peptides.

Our current study also provides new evidence that the timing of application of RM-pull strategy in parenteral vaccine-primed hosts matters and it is the most effective in inducing T_RM_ cells in the lung when it is applied during the memory phase of T cell activation, as opposed to the effector phase of T cell responses. In other words, the recently activated effector T cells recruited into the lung even with cognate Ags do not differentiate into T_RM_ cells as effectively as their effector memory counterparts.

Overall, first of all, our study shows the inability of parenteral vaccine alone to induce T_RM_ cells in the lung. Secondly, it indicates the importance of inclusion of cognate protein-based antigens in RM-pull strategies. Thirdly, it suggests that to be effective, an RM-pull strategy ought to be implemented during the memory phase of T cell responses following parenteral prime immunization. Our study has thus provided new mechanistic insights into the development of CD8 T_RM_ cells in the lung and shall help develop effective parenteral-primed and RM-pull vaccine strategies.

## Data Availability

All datasets generated for this study are included in the manuscript/Supplementary Files.

## Author Contributions

SH, MJ, and ZX conceived and designed the study. SH and MJ analyzed the data. SH, MV-S, YY, SA, MD'A, and AZ performed experiments. SH, MJ, and ZX wrote the manuscript.

### Conflict of Interest Statement

The authors declare that the research was conducted in the absence of any commercial or financial relationships that could be construed as a potential conflict of interest.
