# Supplementary material for: Mucosal-Pull Induction of Lung-Resident Memory CD8 T Cells in Parenteral TB Vaccine-Primed Hosts Requires Cognate Antigens and CD4 T Cells
Source: Front Immunol. 2019 Sep 6;10:2075. doi: 10.3389/fimmu.2019.02075 (PMC6747041; doi:10.3389/fimmu.2019.02075)
Supplement: Supplementary file 1 [file Data_Sheet_1.docx]

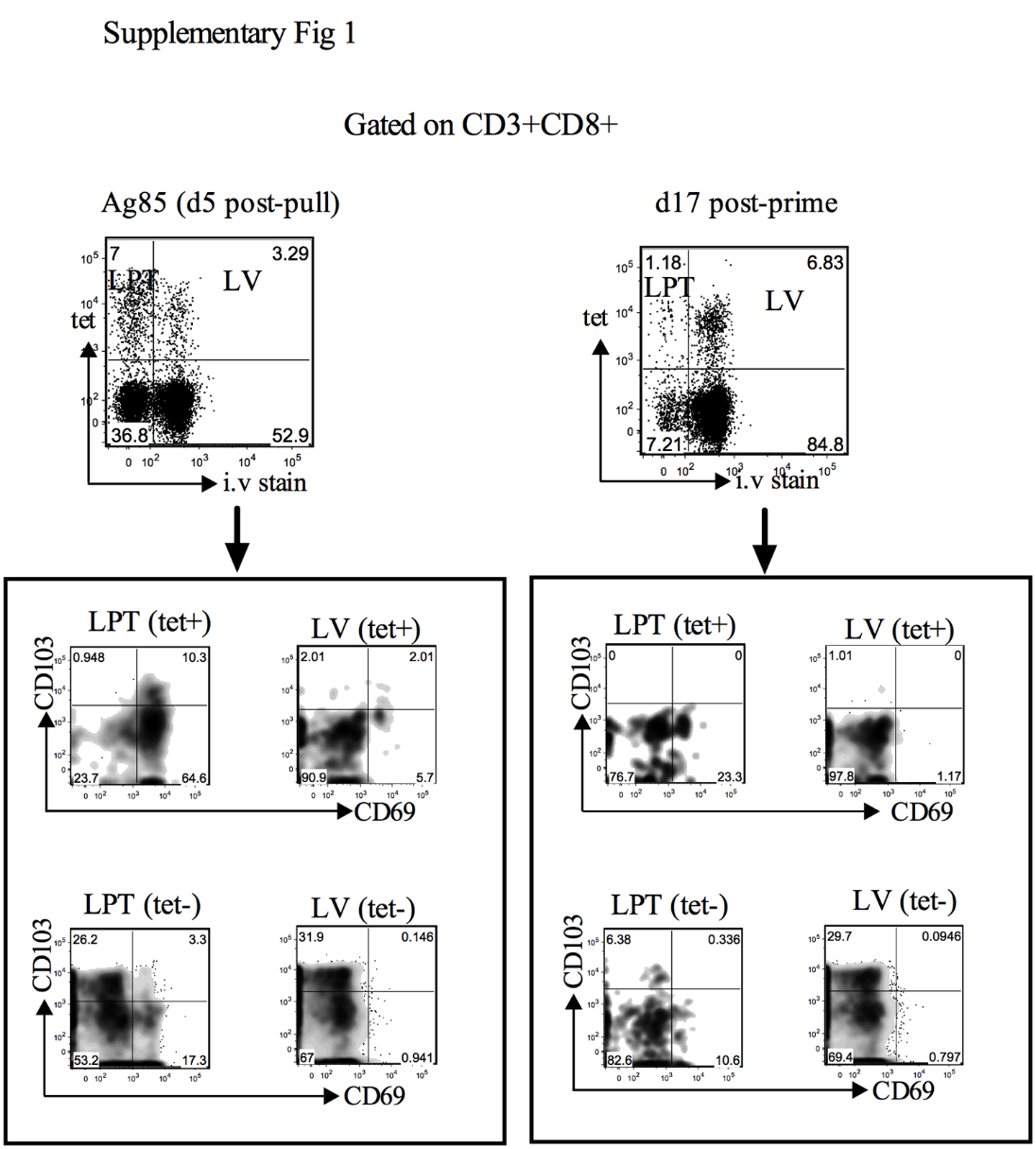


**Supplementary Figure 1. Gating strategy to define T_RM_ cells in the lung parenchymal tissue (LPT) and the lung vasculature (LV).** Experimental design was the same as Figure 1a. Top panel showing cells gated out of CD3+CD8+ population for i.v and tetramer immunostaining in mice primned and Ag85-pulled (left -5d post-pull) and in mice primed only (right -17d post-prime). Bottom panels showing surface expression of CD69 and CD103 markers on tetramer+ and tetramer – cells in LPT and LV of these mice.


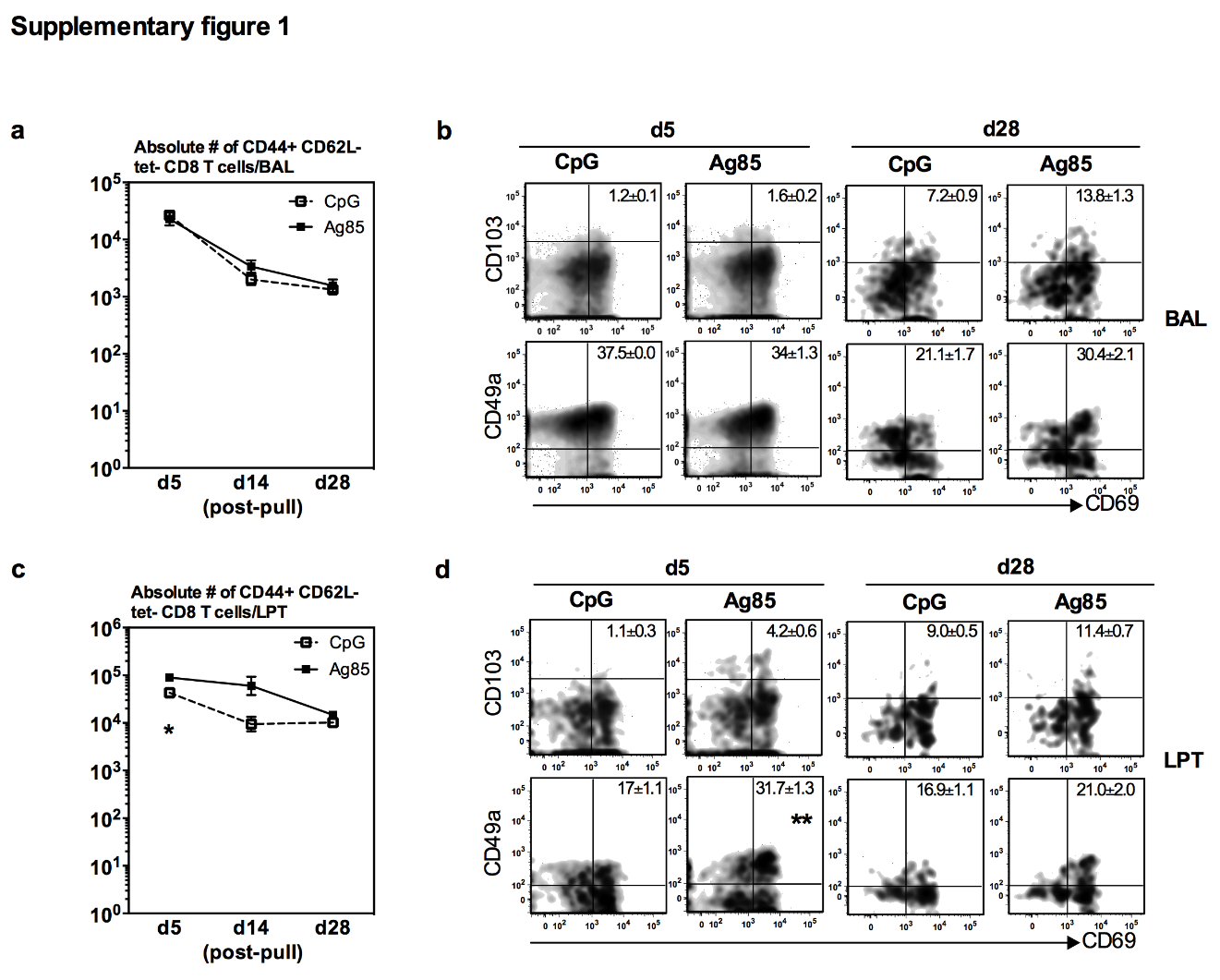


**Supplementary Figure 2. Limited induction of non-Ag-specific CD8 T_RM_ cells by cognate Ag-based RM pull strategy in the effector phase of T cell responses to parenteral immunization.** Experimental design was the same as Figure 1a. (**a**) Line graph showing kinetic changes in numbers of tetramer- CD8 T cells in the airway. (**b**) Representative dotplots showing frequencies of tetramer- CD8 T cells co-expressing CD69 and CD103, or CD69 and CD49a in the airway at day 5 and 28 post-RM pull. (**c**) Line graph showing kinetic changes in numbers of tetramer- CD8 T cells in the lung parenchymal tissue (LPT). (**d**) Representative dotplots showing frequencies of tetramer- CD8 T cells co-expressing CD69 and CD103, or CD69 and CD49a in LPT at day 5 and 28 post-RM pull. Data are expressed as the mean±S.E.M. of three mice/group/timepoint, representative of two independent experiments. *P˂0.05, **P˂0.01 compared with CpG group.


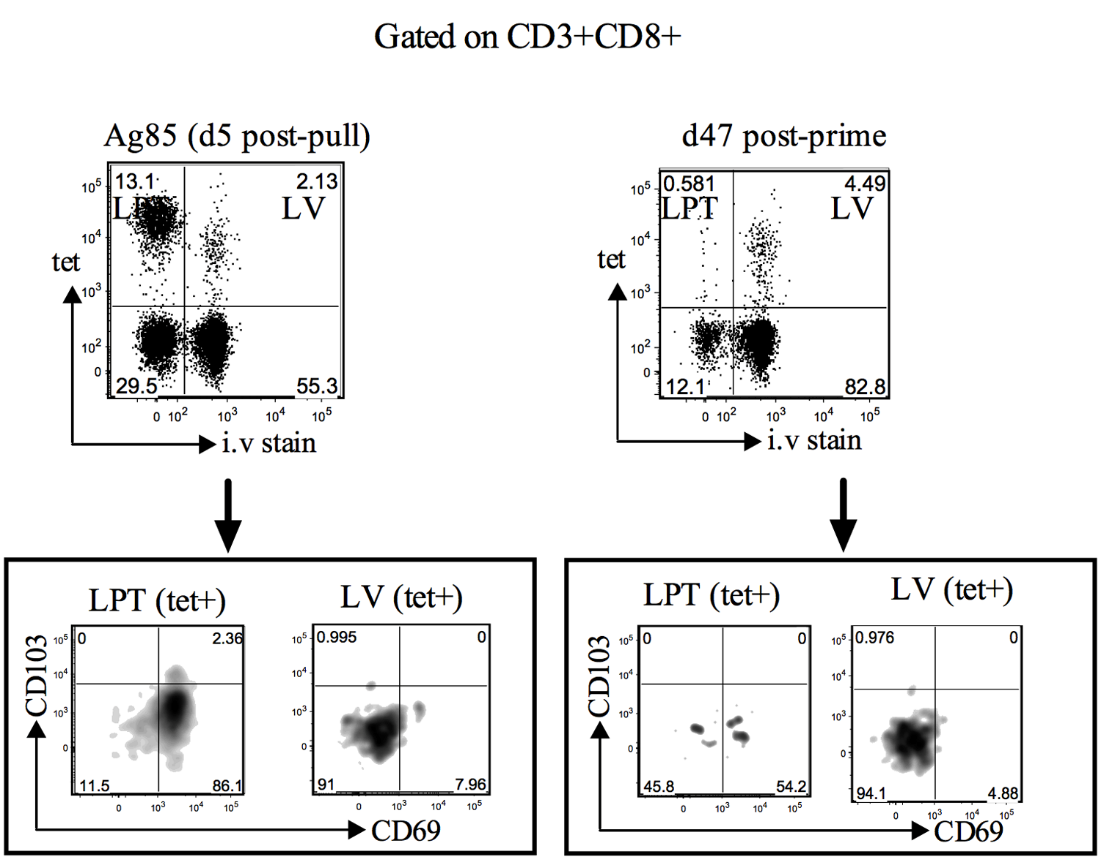


**Supplementary Figure 3. Gating strategy to define T_RM_ cells in the lung parenchymal tissue (LPT) and the lung vasculature (LV).** Experimental design was the same as Figure 2a. Top panel showing cells gated out of CD3+CD8+ population for i.v and tetramer immunostaining in mice primed and Ag85-pulled (left -5d post-pull) and in mice primed only (right -47d post-prime). Bottom panels showing surface expression of CD69 and CD103 markers on tetramer+ cells in LPT and LV of these mice.

**Supplementary Figure 4. Distinct induction of CD8 T_RM_ cells in lung vasculature and lung draining lymph nodes upon cognate Ag-based RM pull in the memory phase of T cell**
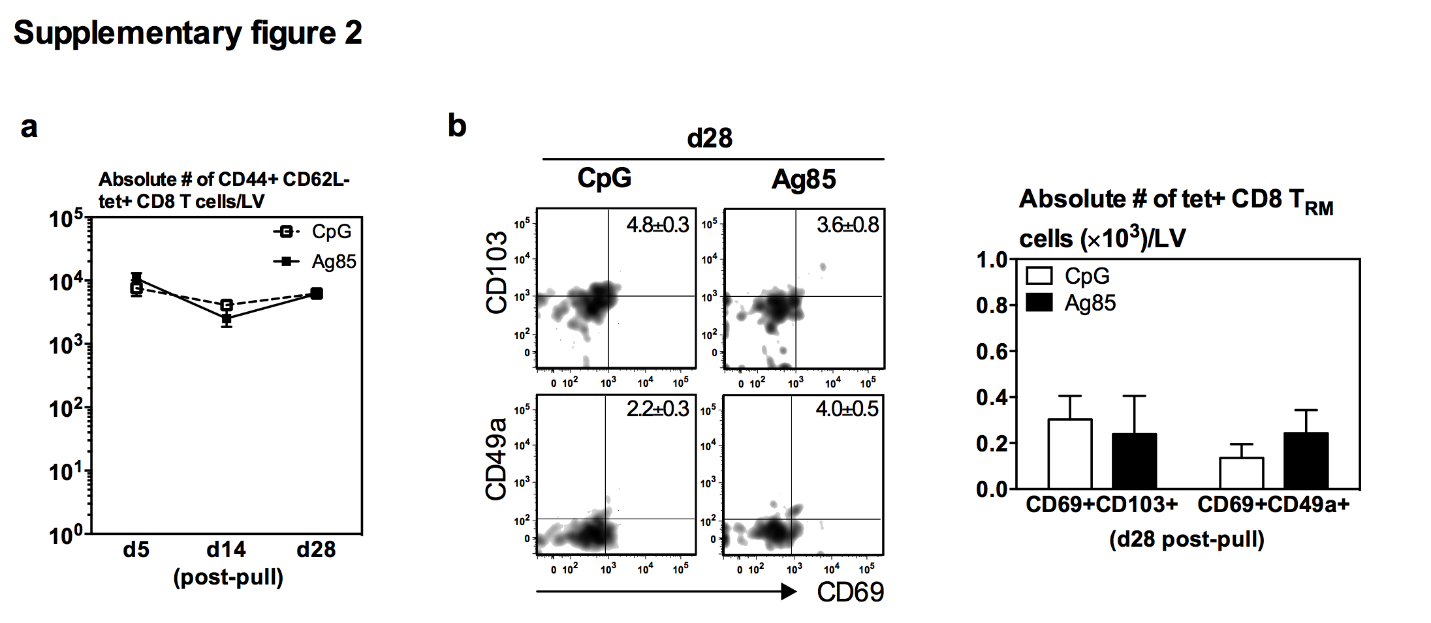
**responses to parenteral immunization.** Experimental design was the same as Figure 2a. (**a**) Line graph showing kinetic changes in numbers of tetramer+ CD8 T cells in the lung vasculature (LV). (**b**) Representative dotplots showing frequencies of tetramer+ CD8 T cells co-expressing CD69 and CD103, or CD69 and CD49a, and bar graph showing numbers of tetramer+ CD8 T cells co-expressing CD69 and CD103, or CD69 and CD49a in LV at d28 post-RM pull. Data are expressed as the mean±S.E.M. of three mice/group/timepoint, representative of two independent experiments. *P˂0.05, **P˂0.01 compared with CpG group.


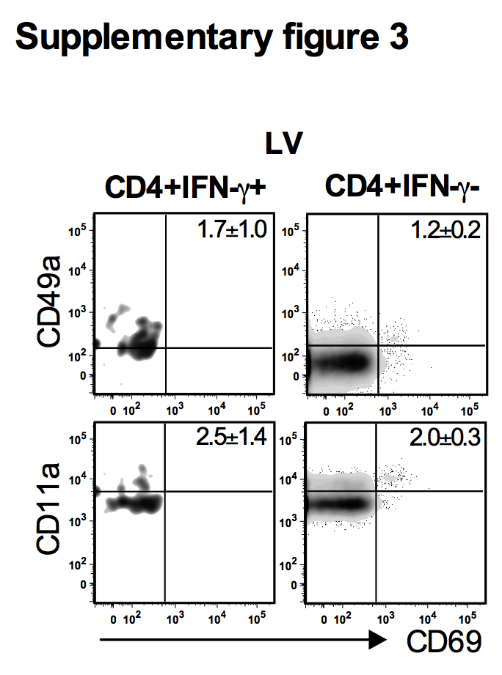


**Supplementary Figure 5. Limited induction of CD4 T_RM_ cells in the lung vasculature upon cognate Ag-based RM pull in the memory phase of T cell responses to parenteral immunization.** Experimental design was the same as in Figure 2a except that BAL and lung mononuclear cells were *ex vivo* re-stimulated with Ag85A CD4 peptide. Representative dotplots showing frequencies of Ag85A-specific interferon-γ (IFN-γ)-producing CD4 T cells co-expressing CD69 and CD49a, or CD69 and CD11a in the lung vasculature (LV) at d28 post-RM pull. Data are expressed as the mean±S.E.M. of three mice/group, representative of two independent experiments.
